# The complete mitochondrial genome of marine gastropod *Sydaphera spengleriana* (Deshayes, 1830) (Neogastropoda: Cancellariidae)

**DOI:** 10.1080/23802359.2021.2013743

**Published:** 2022-02-07

**Authors:** Zongxing Wang, Jingxi Liu, Yuanjin Liu, Zongjun Xu, Fengrong Zheng, Wei Chen

**Affiliations:** a MNR Key Laboratory of Marine Eco-Environmental Science and Technology, First Institute of Oceanography, Ministry of Natural Resources, Qingdao, China; b Shandong Marine Resource and Environment Research Institute, Yantai, China

**Keywords:** Mitogenome, *Sydaphera spengleriana*, phylogenetic analysis

## Abstract

The complete mitochondrial genome of *Sydaphera spengleriana* was characterized for the first time. The total length of the mitogenome was 16,336 bp including a standard set of 13 protein-coding (PCGs), 22 transfer RNA (tRNA), and 2 ribosomal RNA (rRNA) genes. The overall nucleotides base composition of the heavy strand is A (26.44%), G (19.89%), C (13.25%), and T (40.41%), with an A + T bias (66.85%). With the exception of eight tRNA genes, all other mitochondrial genes are encoded on the heavy strand. The mitogenomic phylogenetic relationships indicated that *S. spengleriana* was clustered with *Bivetiella cancellata* within the family Cancellariidae clade with a high bootstrap value.

*Sydaphera spengleriana* (Deshayes, 1830) belongs to Cancellariidae in Neogastropoda (Sowerby 1841; WoRMS 2021). The Cancellariidae is a diverse gastropod family containing ca. 300 living species around the world, which currently inhabiting mainly in tropical and temperate regions, from subtidal to bathyal depths (Petit and Harasewych 2005; Modica et al. 2011; Qu 2019). There is only one complete mitochondrial genome of the species *Bivetiella cancellata* in Cancellariidae recorded in the NCBI database. In this work, we have determined and described for the first time of the complete mitochondrial genome of *S. spengleriana*, which will provide a better insight into phylogenetic assessment and taxonomic classification.

One *S. spengleriana* individual was collected from Laizhou Bay (37.78°N, 119.33°E), China, during August 2020. Specimen (Voucher no. FIO-XLT-JH-002) was deposited in the Biodiversity Lab of the First Institute of Oceanography, MNR. The total genomic DNA was extracted from the muscle of the specimen using a QIAamp Fast DNA Tissue kit (Qiagen, Germany) following the manufacturer’s protocol. DNA libraries (350 bp insert) were constructed with the TruSeq NanoTM kit (Illumina, USA). The complete mitogenome of *S. spengleriana* was sequenced on the Illumina Novaseq 6000 sequencing platform (Illumina, USA) at Science Corporation of Gene, China. The clean data were then assembled using the NOVOPlasty (Dierckxsens et al. 2017). Gene annotation was performed by MITOS (Bernt et al. 2013). The information of this mitochondrial genome was submitted to Genbank and the accession number is MZ934412.

The circular mitochondrial genome of *S. spengleriana* was 16,336 bases long. Its genomic structure is as follows: There are 13 protein-coding (PCGs), 22 transfer RNA (tRNA), and two ribosomal RNA (rRNA) genes, which are the same as the other Cancellariidae species, *B. cancellata* (Cunha et al. 2009). The overall base composition of the mitogenome is estimated to be A 26.44%, T 40.41%, G 19.89%, and C 13.25%, with a high AT bias (66.85%). Most of the genes encoded on the heavy strand, except for the eight tRNA genes (*tRNA-Gln, tRNA-Gly, tRNA-Glu, tRNA-Cys, tRNA-Tyr, tRNA-Met, tRNA-Trp, tRNA-Thr*) encoded on the light strand. Most PCGs begin with ATG except for *nd4* with GTG. The longest gene is *nd5* (1719 bp) among the PCGs whereas the shortest is *tRNA-His* (64 bp). The rRNA genes, 12S rRNA (952 bp) and 16S rRNA (1344 bp) are located between the *tRNA-Met* (CAT) and *tRNA-Leu* (TAG) genes and are separated by the *tRNA-Val* (TAC) gene.

The phylogenetic distance was estimated using the concatenated set of the whole 13 PCGs of *S. spengleriana* mitogenome with 22 published Neogastropoda mitogenomes and *Haliotis rubra* mitogenome as an outgroup (Figure 1).

**Figure 1.**
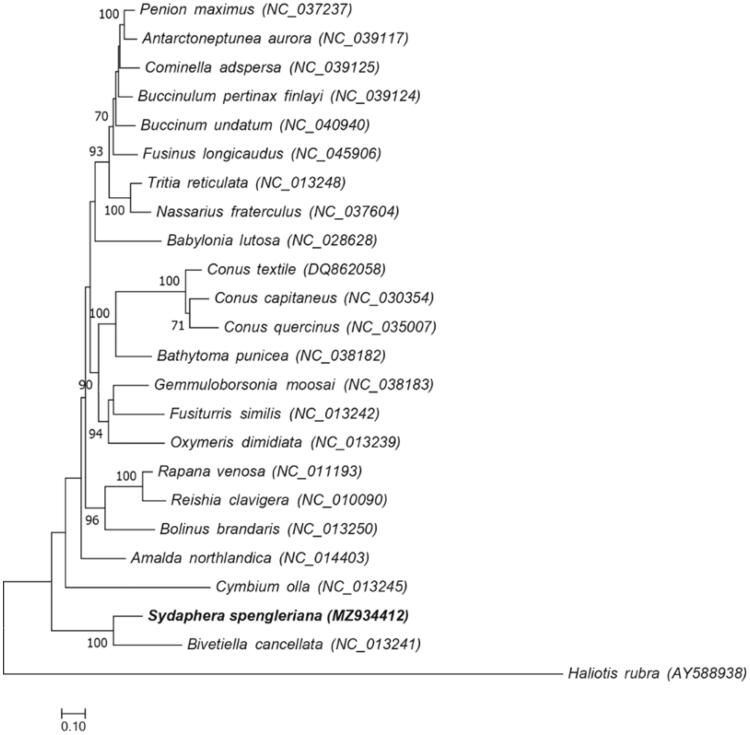
Maximum-likelihood tree showing phylogenetic relationships among 23 Neogastropoda species. Sequences from *Haliotis rubra* (AY588938) is used as outgroup. Numbers in the nodes correspond to ML bootstrap proportions. Only values above 70% are represented.

The phylogenetic analysis indicated that *S. spengleriana* was clustered with *B. cancellata* within the family Cancellariidae. Phylogenetic relationships within Neogastropoda were consistent with the phylogenetic analyses of Neogastropoda based on the entire Mitogenome (Cunha et al. 2009). Our data offered useful information for the studies of *S. spengleriana* on evolutionary characteristics, phylogenetic relationships as well as species identification.

## Data Availability

The genome sequence data are deposited in GenBank of NCBI at https://www.ncbi.nlm.nih.gov/nuccore/under the accession no. MZ934412. The associated BioProject, SRA, and Bio-Sample numbers are PRJNA761868, SRR15836237, and SAMN21365079, respectively.

## References

[CIT0001] Bernt M, Donath A, Jühling F, Externbrink F, Florentz C, Fritzsch G, Pütz J, Middendorf M, Stadler PF. 2013. MITOS: improved de novo metazoan mitochondrial genome annotation. Mol Phylogenet Evol. 69(2):313–319. 22982435 10.1016/j.ympev.2012.08.023

[CIT0002] Cunha RL, Grande C, Zardoya R. 2009. Neogastropod phylogenetic relationships based on entire mitochondrial genomes. BMC Evol Biol. 9:210. 19698157 10.1186/1471-2148-9-210 PMC2741453

[CIT0003] Dierckxsens N, Mardulyn P, Smits G. 2017. NOVOPlasty: de novo assembly of organelle genomes from whole genome data. Nucleic Acids Res. 45(4):e18. 28204566 10.1093/nar/gkw955 PMC5389512

[CIT0005] Modica MV, Bouchet P, Cruaud C, Utge J, Oliverio M. 2011. Molecular phylogeny of the nutmeg shells (Neogastropoda, Cancellariidae). Mol Phylogenet Evol. 59(3):685–697. 21440647 10.1016/j.ympev.2011.03.022

[CIT0006] Petit RE, Harasewych MG. 2005. Catalogue of the superfamily Cancellarioidea. Forbes & Hanley, 1851 (Gastropoda: Prosobranchia), 2nd edition. Zootaxa. 1102(1): 1–161.

[CIT0007] Qu XC. 2019. Zhongguo Changjian Haiyang Shengwu Yuanse Tudian: Ruanti Dongwu. Qingdao (China): China Ocean University Press; p. 218.

[CIT0008] Sowerby GBI. 1841. *Cancellaria*. In: The conchological illustrations. London (UK): Sowerby.

[CIT0009] WoRMS. 2021. Sydaphera spengleriana (Deshayes, 1830); [accessed 2021 September 13]. https://www.marinespecies.org/aphia.php?p=taxdetails&id=464837

